# Analysis of Magnetic Anisotropy and Non-Homogeneity of S235 Ship Structure Steel after Plastic Straining by the Use of Barkhausen Noise

**DOI:** 10.3390/ma13204588

**Published:** 2020-10-15

**Authors:** Martin Jurkovič, Tomáš Kalina, Katarína Zgútová, Miroslav Neslušan, Martin Pitoňák

**Affiliations:** 1 The Faculty of Operation and Economics of Transport and Communications, University of Žilina, Univerzitná 1, 010 26 Žilina, Slovakia; tomas.kalina@fpedas.uniza.sk; 2 Faculty of Civil Engineering, University of Žilina, Univerzitná 1, 010 26 Žilina, Slovakia; katarina.zgutova@fstav.uniza.sk (K.Z.); martin.pitonak@fstav.uniza.sk (M.P.); 3 Faculty of Mechanical Engineering, University of Žilina, Univerzitná 1, 010 26 Žilina, Slovakia; miroslav.neslusan@fstroj.uniza.sk

**Keywords:** Barkhausen noise, S235 steel, plastic straining

## Abstract

This study investigates the microstructure, residual stress state, and the corresponding magnetic anisotropy of the ship structure samples made of S235 steel after uniaxial tensile deformation. A non-destructive magnetic technique based on Barkhausen noise is employed for fast and reliable monitoring of samples exposed to the variable degrees of plastic straining. It was found that the progressively developed plastic straining of the matrix results in an alteration of the easy axis of magnetization, stress anisotropy (expressed in residual stresses state) as well as the corresponding Barkhausen noise emission. Moreover, remarkable non-homogeneity can be found within the plastically strained region, especially when the localized plastic straining takes place.

## 1. Introduction

The development and promotion of green transport solutions has resulted in an increased demand for shipbuilding. New design solutions, increasing demands for reliability, efficiency, and cost-effectiveness of ships, require new ways of ensuring these attributes [1]. Among the most serious structural problems of the structural design are fatigue damage and corrosion. External influences on ship structures (waves, cargo, etc.), as well as civil structures, cause constant structural stress, which results in the accumulation of fatigue damage in ship structures [2].

Furthermore, the corrosion extent in seagoing ships and civil structures, as a result of an aggressive environment, can negatively affect their effective cross sectional areas and contribute to their over-stressing. Rupture of bodies can occur as a result of highly developed plastic straining. Breakdown in a certain region of shipbuilding or civil structures can also redistribute the exerted load and increase the true stress into the neighboring regions [3]. Over-stressing (or rupture) in the localized region could initiate an unexpected collapse. For this reason, several techniques have been developed for assessment of the corrosion or/and fatigue extent and the associated over-stressing [4,5,6]. Wang et al. [7] developed the model approach for assessment of the corrosion extent in steel structures. Over-stressing is usually connected with plastic straining and the corresponding alteration of microstructure. The main mechanism contributing to the plastic straining is the increased dislocation density and the corresponding increase in the matrix hardness. However, there should be a distinction between homogenous plastic strain and necking, when the exerted loading energy is localized in a narrow region. Non-homogenous plastic straining (necking) alters the incoming rupture of a body and represents the final phase of over-stressing. Suitable non-destructive, fast, and portable testing methods employed for such a purpose could be beneficial in order to reveal the critical regions of shipbuilding and civil constructions.

The main parts of shipbuilding and civil constructions are made of conventional steels. These steels are entirely composed of ferromagnetic ferrite or ferrite-pearlite grains, and magnetic methods can be employed for the aforementioned purpose. Magnetic Barkhausen noise (MBN) is a physical process involving irreversible, and discontinuous, domain walls’ (DWs) motion when the external magnetic field alternates with time. Various lattice defects preclude DWs in their position and their sudden jumps occur as soon as the strength of the magnetic field attains the critical threshold equal to the pinning strength of the pinning sites [8,9]. It is believed that MBN would be nearly zero in a completely perfect lattice, and increasing density of lattice imperfections would increase MBN. However, beyond the certain threshold MBN would decrease along with increasing density of lattice imperfections. It is considered that MBN drops down when the density of lattice imperfections (expressed in many terms) increases in the case of conventional ferrite steels (such as S235), since we are far beyond the aforementioned threshold. MBN could increase along with increasing density of lattice imperfections only in the case when the remarkable crystallographic texture and the corresponding DWs alignment takes place and dominates. MBN can be affected by stress. Tensile stress increases the number of the produced electromagnetic pulses during DWs motion, whereas compressive stress tends to decrease MBN [10]. This behavior is due to the alignment of DWs, which is a function of the stress state, as has been explained in previous publications [11,12]. On the other hand, the microstructure also strongly affects MBN. Lattice defects in conventional ferrite steels, more or less, contribute to a decrease in MBN. Expressed in other words, an increasing density of lattice defects means lower MBN. DWs in motion interfere with these lattice defects. For this reason, MBN is sensitive to the microstructural alterations since the MBN signal contains information about collisions of DWs with lattice defects such as dislocation cells [13,14,15], the size and distribution of precipitates [15,16], the density of grain boundaries, and/or non-ferromagnetic phases [6,17]. However, their superimposed contribution to the MBN is usually difficult to unwrap.

Plastic straining significantly alters the stress state (expressed in terms of residual stresses), as well as the microstructure (especially dislocation density and the corresponding microhardness). For this reason, MBN could be a suitable technique for assessment of S235 steel over-stressing. The pilot study focused on this topic has been already reported [18]. This paper reported about comparison between the loaded (in-situ) and unloaded samples. This study deals with the influence of the progressively developed plastic straining on the MBN emission, which is correlated with residual stresses and dislocation density expressed in terms of microhardness.

## 2. Materials and Methods

**Figure 1:**
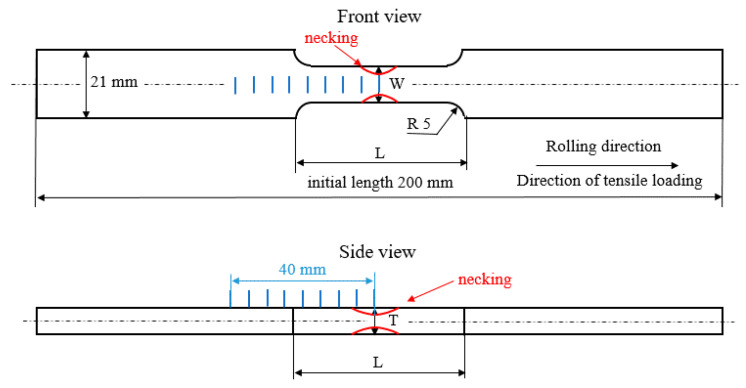
Brief sketch of the samples and positions of the analyzed points, initial *L* = 49.80 mm, *T* = 7.84 mm, *W* = 12.41 mm, distance between the neighboring blue lines 5 mm.

**Figure 2:**
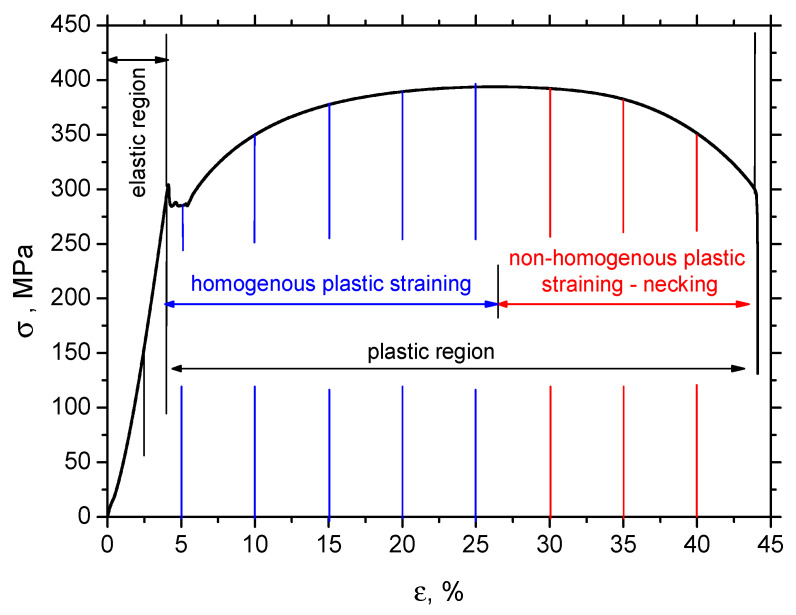
Stress–strain (engineering) curve of S235 steel, deformation speed 1 mm·min^−1^ (strain rate 0.33 × 10^−3^).

**Figure 3:**
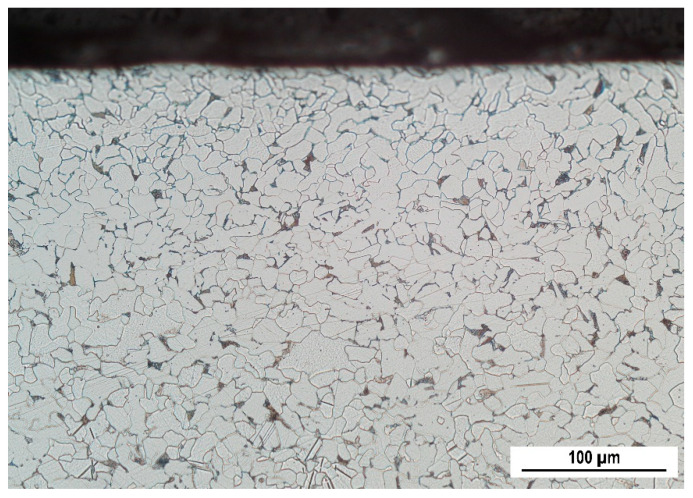
Ferrite matrix (white) with limited volume of pearlite islands (dark), as received.

S235 steel samples (hardness 182 ± 11 HV0.5, yield strength 305 MPa, ultimate strength 390 MPa, and elongation at break 41.3%, hot rolled—as received), as illustrated in Figure 1, were subjected to uniaxial tensile loading by the use of an Instron 5985 (Instron, Norwood, MA, USA). The direction of the tensile stressing corresponds with the rolling direction (RD). The transverse direction is referred to as TD. Chemical composition of S235 is indicated in Table 1. The samples were investigated after loading (in the unloaded state). Samples were subjected to the predefined plastic straining, as shown in Figure 2 and Table 2. The as-received microstructure of the S235 steel is entirely composed of ferrite grains and neighboring small pearlite islands (see Figure 3). MBN, XRD (X-ray diffraction), and microhardness measurements were carried out in the sample center as well as in the predefined points, as shown in Figure 1 (indicated by blue lines—the distance between the neighboring points was kept a constant 5 mm). The preliminary phase of experiments revealed that the evolution of sample state is symmetrical along the length with respect to the sample center.

Plastic deformation tends to reduce the cross sectional area (expressed in terms *W* × *T*, see Figure 1) and makes the samples longer (expressed in term *L*, see Figure 1). For this reason, the sample’s dimensions after loading were measured by the use of a caliper measure (precision ± 0.01 mm) in the sample center. The measured values of *L*, *W*, and *T* are shown in Table 2. This table also compares engineering *σ* and true stress *σ_true_* calculated by the use of *T* and *W* values. *T* and *W* values were found to be nearly constant within the whole *L* after the homogenous plastic straining. On the other hand, *T* and *W* values refer to the dimensions of the neck (indicated by a red color in Figure 1) when the non-homogenous plastic straining takes place. The distance between the neighboring positions (indicated by blue color in Figure 1) was kept constant at 5 mm and drawn after the sample unloading. Due to elongation of the region defined by *L* during tensile test the number of positions within this region increased from 5 (for the lower strains) to 6 as soon as the strains exceeded 20%. It is also worth to mention that MBN technique indicated altered state of the sample in the region in which the radius R5 occurs (see Figure 1) and beyond the region defined by *L* distance (outside the thinned region) as well.

The true interpretation of MBN signals is associated with the investigation of the interrelated relationships among the MBN, residual stresses, and microstructure. For this reason, the non-destructive XRD measurements, conventional destructive metallographic observations (light microscopy), and microhardness readings were also carried out. Microstructural observations were carried out in the sample’s center in the RD direction with respect to their length, as well as width. The specimens of the length 20 mm were cut by the use of a Struers Secotom-50 (Struers Inc., Cleveland, OH, USA) and routinely prepared for metallographic observations (hot molded, ground, polished, and etched by 3% Nital for 10 s).

Microhardness (HV0.5) was measured by the use of an Innova Test 400^TM^ device (Innovatest, Maastricht, The Netherlands)—loading force 500 g for 10 s. Microhardness values were obtained by the average of three repetitive measurements at the nine different points along the sample length, as illustrated in Figure 1 (blue lines).

Residual stresses were determined from XRD patterns measured by a Proto iXRD Combo diffractometer (Proto manufacturing Ltd., Oldcastle, ON, Canada)—Cr*Kα* radiation, the effective penetration depth ~5 µm, scanning angle ± 39°, and Bragg angle 156.4°. The residual stresses were calculated from shifts of the 211 reflection. The Winholtz and Cohen method and X-ray elastic constants *½s_2_* = 5.75 TPa^−1^, *s_1_* = −1.25 TPa^−1^ were used for residual stress determination. Residual stresses were determined from the diffraction patterns by calculating the strain from the diffraction peak positions. X-ray diffraction measures the distance between crystallographic planes. This distance is obtained from the diffraction angle 2θ and the known X-ray wavelength using Bragg’s Law. Residual stresses were measured in the RD as well as the TD directions for the predefined plastic strains. Nine different points along the sample were measured, as illustrated in Figure 1 (blue lines).

**Figure 4:**
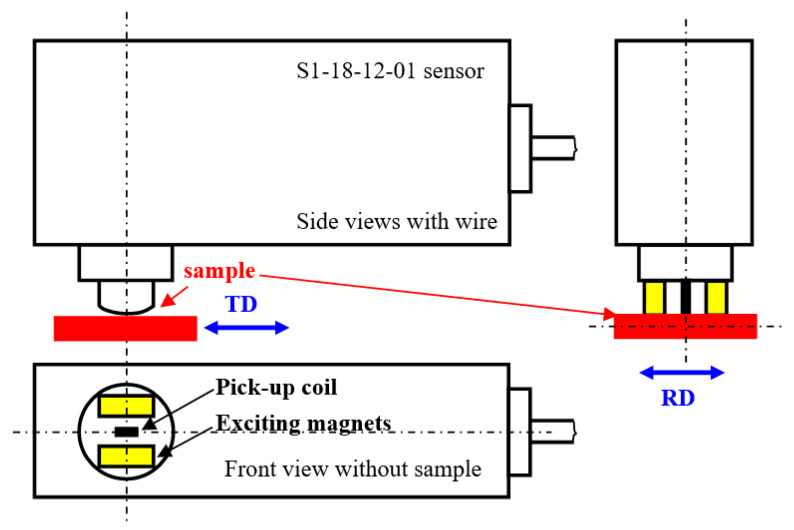
Brief sketch of the experimental set-up.

**Figure 5:**
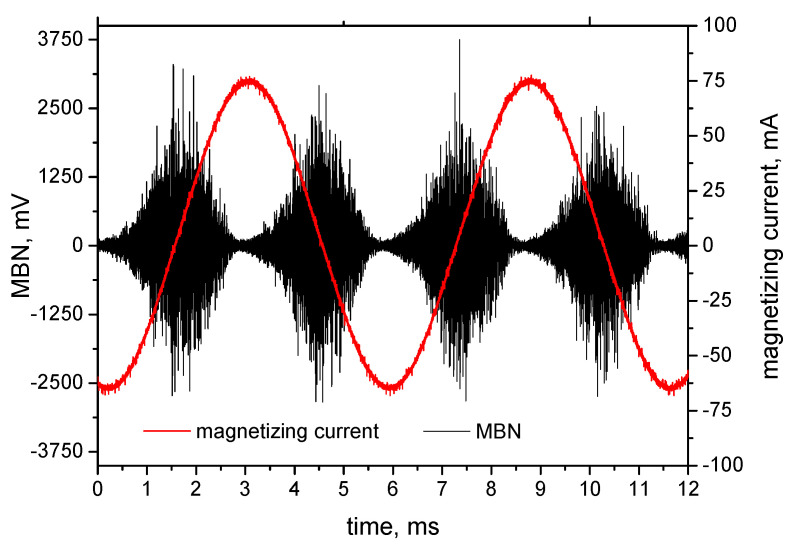
Magnetic Barkhausen noise (MBN) signal measured in rolling direction (RD) for *ε* = 2.5%.

MBN was measured after unloading by the use of a RollScan 350 (Stresstech, Jyväskylä, Finland) in the RD as well as TD directions. MBN emission was measured for the predefined plastic strains. Nine different points along the sample were investigated, as illustrated in Figure 1 (blue lines). The high frequency MBN signal was measured by the S1-18-12-01 sensor with a sine waveform generator (magnetizing voltage 3.5 V and frequency 175 Hz, frequency range of MBN emission from 20 up to 700 kHz, sampling frequency 6.7 MHz, estimated MBN sensing area about 4 mm^2^, estimated skin depth in the range from 200 ÷ 300 µm [19,20]) and processed using MicroScan 600 software (Stresstech, Jyväskylä, Finland). Magnetizing voltage is the amplitude of voltage (of the sine waveform) on the exciting magnets during the sample magnetizing. This voltage corresponds to the magnetizing current in the sample of amplitude 62 mA. MBN represents the effective (*rms*) value of the MBN emission. The peak position (*PP*) and the number of MBN pulses were also extracted from the raw MBN signal. *PP* refers to the strength of the magnetic field in which the MBN envelope attains the maximum. Number of MBN pulses was measured by MicroScan software when each detected pulse exceeding the threshold was counted (the floating threshold applied). A brief sketch of the experimental set-up is illustrated in Figure 4. The example of MBN signal measured in RD for *ε* = 2.5% is shown in Figure 5.

## 3. Experimental Results

### 3.1. Light Microscopy and Microhardness

**Figure 6:**
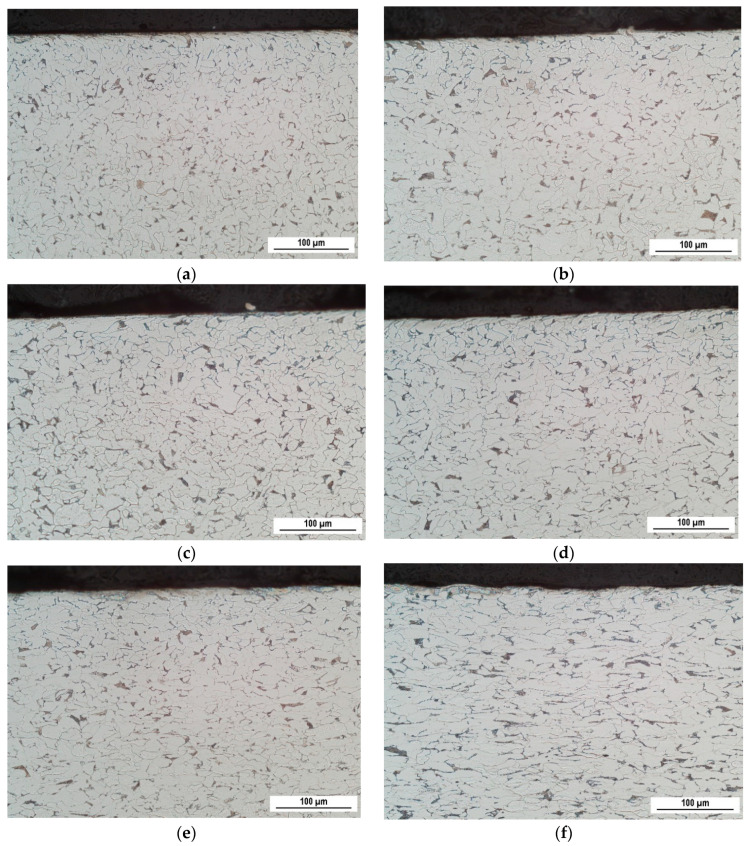
Optical images for the different ε. (**a**) *ε* = 5%; (**b**) *ε* = 15%; (**c**) *ε* = 20%; (**d**) *ε* = 30%; (**e**) *ε* = 35%; (**f**) *ε* = 40%.

The microstructure of S235 corresponds to the chemical composition indicated in Table 1. Due to the low volume of carbon, the steel matrix is mostly composed of ferrite grains (appearing white in Figure 6) and small neighboring pearlite islands of limited size (appearing black in Figure 6). The ferrite grains are equiaxed and their remarkable elongation, due to severe plastic deformation, is apparent for the highest plastic strains only (see Figure 6). Typical preferential orientation in the RD is a result of the elongation of grains along the exerted load, which is asymmetrical (uniaxial tensile stress). It can also be reported that the remarkable preferential elongation becomes more apparent as soon as the homogenous plastic straining is replaced by the localized one (as soon as the plastic strain exceeds 25%). Apart from the predominant ferrite, the minor phase—pearlite—also undergoes preferential plastic straining in the RD. The microstructure for the localized plastic strains was observed in the position of the produced neck.

Apart from the preferential elongation of the S235 steel matrix, plastic deformation also alters the dislocation density and the corresponding microhardness. Plastic straining in S235 occurs due to the opposition of neighboring dislocations. The free path of dislocation motion is followed by its interference with other dislocations (dislocation cells) and their intersections. Therefore, increasing dislocation density also increases the shear stress due to increasing opposition from the dislocation generated in the previous phase of plastic deformation. This mechanism increases the tensile stress necessary to develop the higher *ε*, as Figure 1 demonstrates. This mechanism is also valid for the non-homogenous phase of plastic straining. The engineering stress shows a remarkable decrease in *σ* in Figure 1. However, the true stress *σ_true_* (calculated on the basis of *T*, *W* values indicated in Table 2) clearly demonstrates progressive growth within the whole range of plastic deformation.

**Figure 7:**
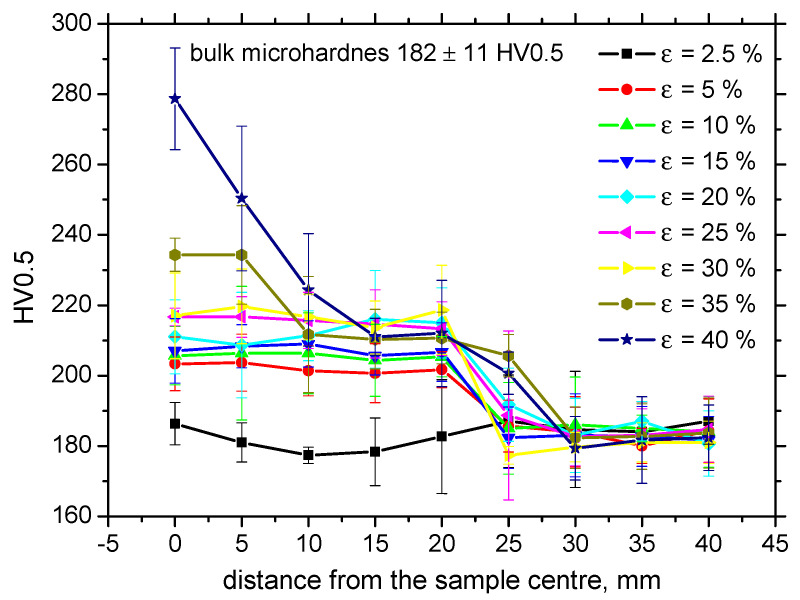
Evolution of microhardness along the sample length as a function of *ε*.

**Figure 8:**
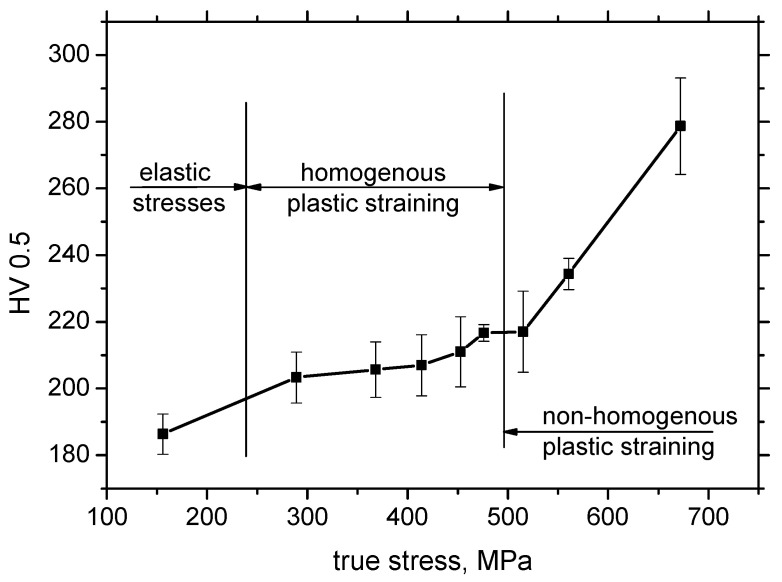
True stresses versus HV0.5, distance from the sample center 0 mm.

Increasing dislocation density can be expressed in terms of microhardness, as measured for various plastic strains as well as positions along the sample length (see Figure 7). Microhardness for the sample loaded in the elastic region (*ε* = 2.5%) corresponds to the bulk microhardness, since the elastic regime does not initiate any dislocation slip; therefore, dislocation density remains untouched. As soon as the yield strength is exceeded, the microhardness increases within length *L* and grows progressively, along with the degree of the homogenous plastic straining. Figure 7 also illustrates that the microhardness is kept nearly constant (in the region of homogenous plastic straining) with respect to the length *L* and drops down to the bulk microhardness at a distance of 25 mm. It can also be reported that the progressive growth of the microhardness in the region of homogenous plastic straining is only moderate, whereas a quite steep increase can be found for the highest plastic stains (*ε* = 35% and 40%, also see Figure 8). Despite the visible decrease in engineering stresses in the phase of non-homogenous plastic straining (indicated in Figure 2), the microhardness increases, since the microhardness is more a function of true stresses in the necked zone, which increases, as Figure 8 clearly demonstrates. Figure 5 also shows that the microhardness drops down for *ε* = 35% and 40%, with respect to the sample length, to the microhardness more or less corresponding to the microhardness developed during the region of homogenous plastic straining and finally falls down to the bulk microhardness along with the further increase in the distance from the sample center. It is also clear that the decrease to the bulk microhardness for *ε* = 35% and 40% is delayed since the position at the distance 25 mm from the sample center becomes positioned within the L region due to the remarkable elongation of the samples (see also the *L* values indicated in Table 2). The high hardness in the sample center for *ε* = 35% and 40% corresponds to the position of the necking and the steep increase in microhardness is due to the remarkable concentration of exerted energy in this narrow zone, as compared with the homogenous regime, where this energy is more or less homogenously distributed within the L region.

### 3.2. XRD Measurements

**Figure 9:**
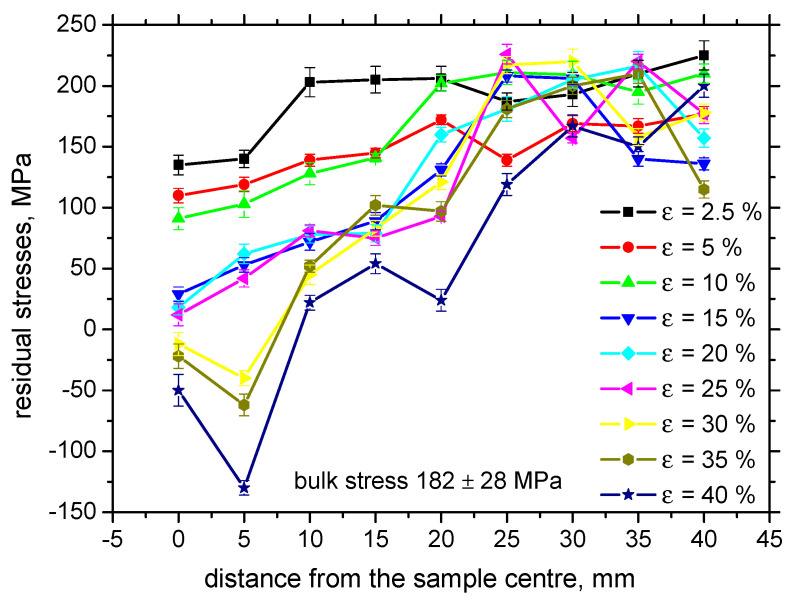
Evolution of residual stresses for the different *ε*, RD.

**Figure 10:**
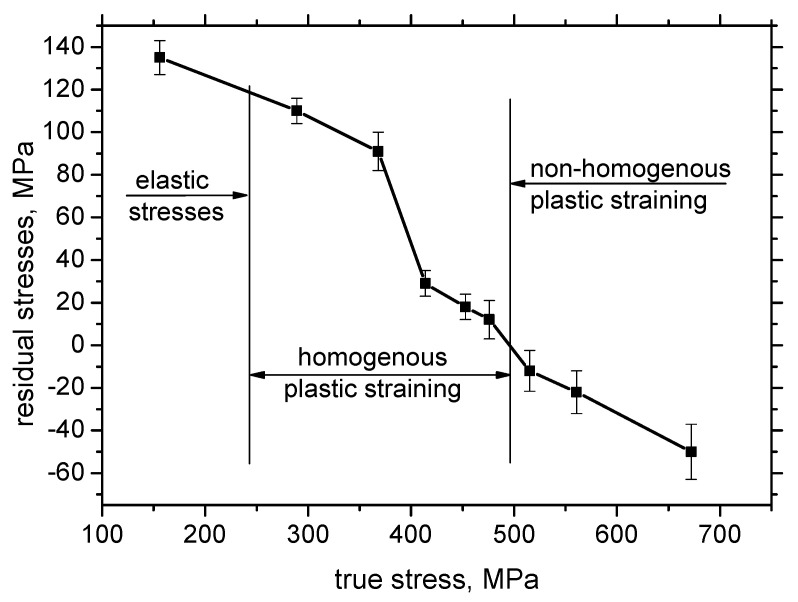
True stresses versus residual stresses.

**Figure 11:**
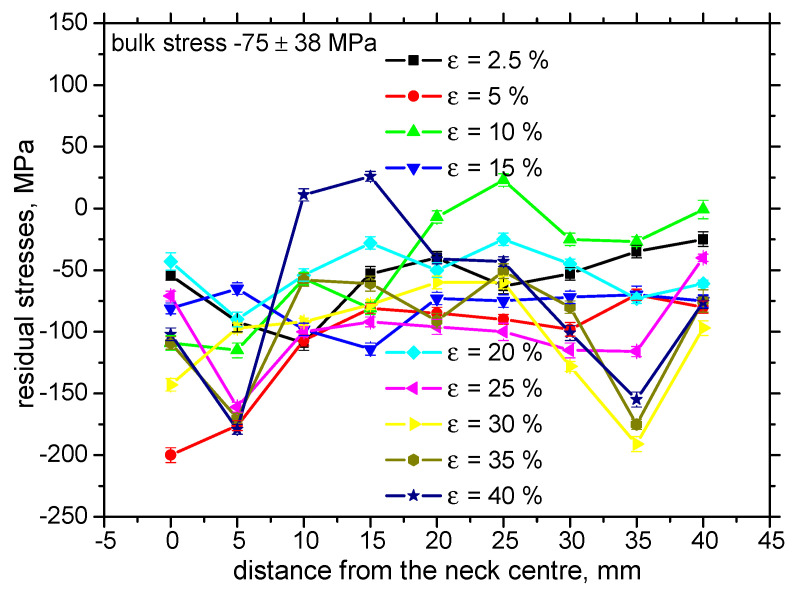
Evolution of residual stresses for the different *ε*, transverse direction (TD).

Figure 9 illustrates that the tensile stresses during loading tend to shift the residual stresses towards the stress free state for the highest *ε* in the region of homogenous plastic straining. Residual stresses are also altered for the elastic regime of loading (*ε* = 2.5%). Increasing true stresses progressively drops down tensile stresses followed by an increasing magnitude of compressive stresses for the non-homogenous plastic straining (Figure 10). As compared with the evolution of HV0.5, the residual stress profiles exhibit progressive growth towards the bulk tensile stress, of about 180 MPa, along the increasing distance from the sample center. Furthermore, the maximum compressive stresses for *ε* = 30%, 35%, and 40% were not found at the exact sample center, but in the neighboring region. Figure 11 illustrates that the correlation in TD was not observed with respect to *ε* as well as with the distance along the sample center.

### 3.3. MBN Analysis

**Figure 12:**
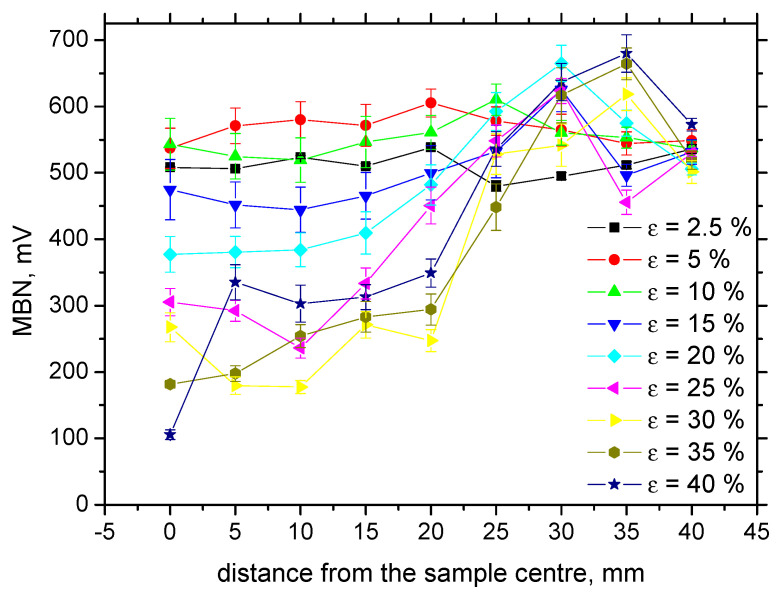
Evolution of MBN along with ε, RD.

Increasing dislocation density, expressed in terms of microhardness, as well as the progressive shift of residual stresses towards the compressive region, contributes to the decreasing MBN. It is well known that tensile stresses align DWs into the direction of these stresses (this alignment increases magnitude of MBN pulses and the effective value of MBN signal), whereas compressive stresses tend to align DWs in the direction perpendicular to the stress [10], which in turn, decreases the magnitude of MBN pulses. Brief illustration of this phenomenon was reported by Karpuschewski et al. [21]. Such behavior occurs when the energy of magnetocrystalline anisotropy is more than magnetoelastic energy [9] (the energy of magnetocrystalline anisotropy is a function of magnetocrystalline anisotropy, whereas the magnetoelastic energy is a function of the isotropic magnetostriction). Increasing dislocation density increases the opposition against the DWs motion. On one hand, dislocation tangles, as pinning sites, preclude the DWs irreversible motion [14,22,23,24]. As soon as the pinning strength of dislocations is exceeded and DWs motion is initiated, the dislocations make shorter the free path of DWs motion, which in turn, also decreases MBN. The particular contributions of the microhardness and residual stresses to the MBN decrease is difficult to unwrap; however, due to their superimposing effect, MBN versus *ε* drops down progressively, especially in the sample center. The distribution of MBN along with the increasing distance from the sample center is less straightforward. As soon as the necking takes place, the drop down to bulk MBN for the highest plastic strains is delayed with respect to the sample length, as compared with medium or low *ε*. Expressed in other words, higher number of the measured points (of constant distance 5 mm) fall into thinned region (defined by *L*) due to the sample larger elongation. Therefore, MBN increases towards the MBN typical for bulk later with respect of the distance from the sample center. Figure 12 also shows that MBN for *ε* = 5% and 10% is more than that for the sample loaded in the elastic regime (*ε* = 2.5%). This behavior has already been explained in terms the non-homogeneity of plastic deformation. Kleber and Vincent [13], as well as Feaugas [25], reported that the intergranular stresses first appear beyond the yielding when the grains, which are of favorable orientation (against the direction of exerted load) are plastically deformed, whereas the others are only elastically deformed. This non-homogeneity from grain to grain predominates in the regions of low *ε* and, remarkably, contributes to the higher MBN as compared to the surface loaded in the elastic regime. As soon as the intragranular stresses become prevalent and spatial heterogeneity of dislocation distribution occurs, MBN versus *ε* drops down after unloading. It is worth mentioning that the shape factor contributes to the decrease in MBN for *ε* = 40% as well, since the necked region in this case is not flat but U-shaped.

**Figure 13:**
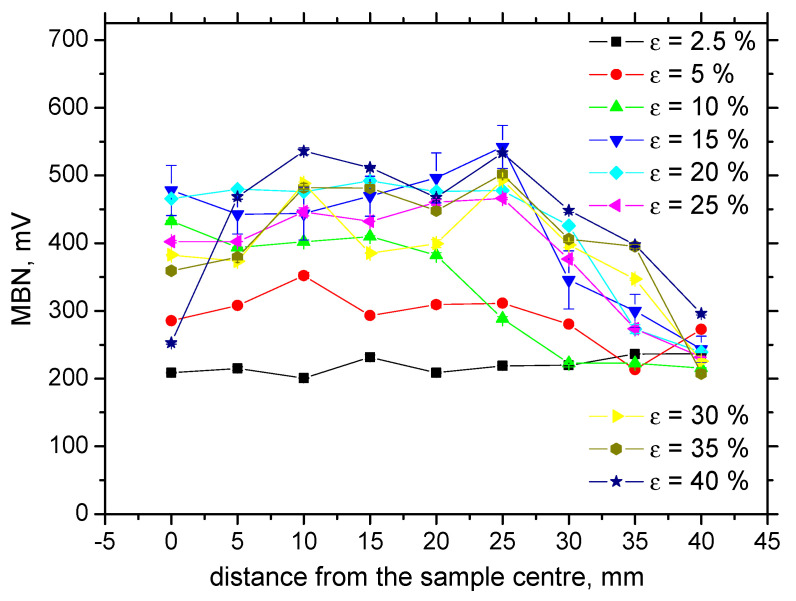
Evolution of MBN along with *ε*, TD.

Evolution of MBN in the TD differs from that in the RD (see Figure 13). MBN grows in the initial phases beyond the yielding followed by early saturation for more developed *ε*. The region *L,* undergoing plastic deformation, exhibits higher MBN that those lying outside of this region. It can also be found that above *ε* = 15%, MBN in the TD are more than in the RD. Expressed in other words, the easy axis of magnetization tends to shift from the RD to TD for more developed *ε*.

**Figure 14:**
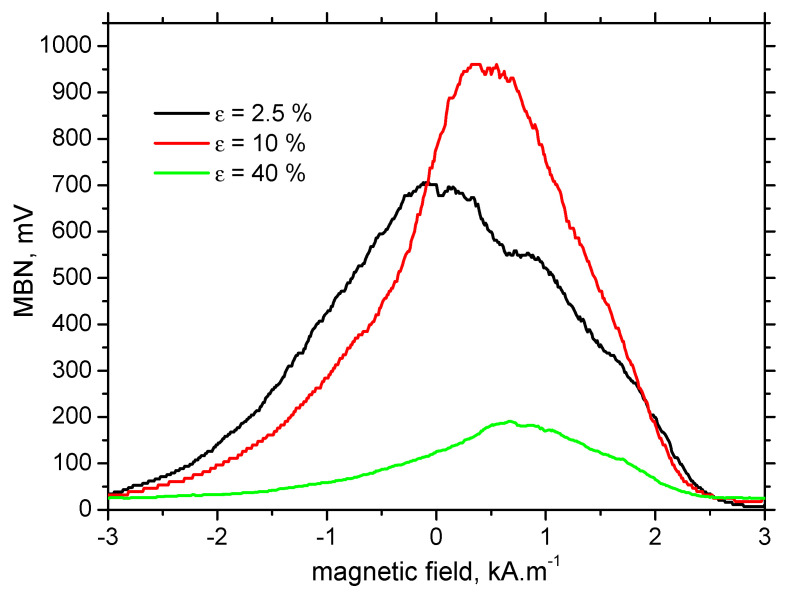
MBN envelopes for the different *ε*, RD.

**Figure 15:**
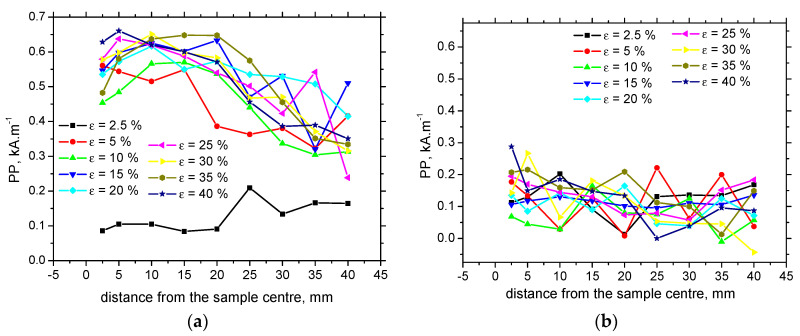
Peak point (PP) versus *ε*. (**a**) PP versus *ε*, RD; (**b**) versus *ε*, TD.

Apart from MBN, MBN envelops and the corresponding *PP* versus *ε* are altered as well. Figure 14 shows that MBN and the corresponding height that the MBN envelopes grow for *ε* = 10% as compared with *ε* = 2.5%, followed by a remarkable decrease for the more developed *ε*. Moreover, these envelopes are shifted towards the stronger magnetic fields in the RD, as Figure 14 and Figure 15a depict. Such an increase is due to increasing opposition of dislocation cells, which hinder DWs’ motion, as well as the contribution of compressive stresses [10,14,23]. As soon as the yielding takes place, *PP* in the RD increases steeply as opposed to the TD. *PP* in the RD decreases along the increasing distance from the sample center. However, the sensitivity of *PP* against *ε* is quite weak. On the other hand, *PP* in the TD exhibits no sensitivity as a function of either *ε* or sample length (see Figure 15b).

**Figure 16:**
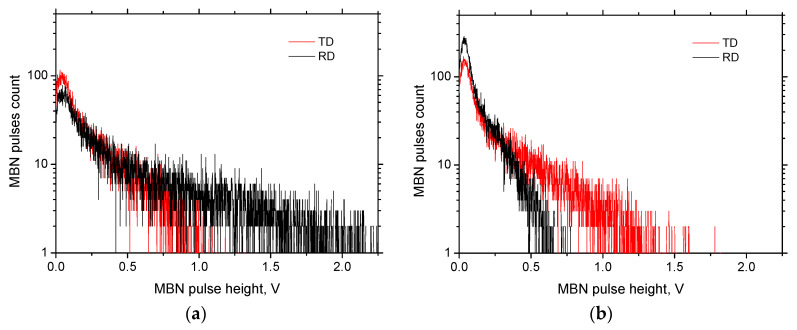
Distribution of MBN pulses height. (**a**) *ε* = 2.5%; (**b**) *ε* = 40%.

**Figure 17:**
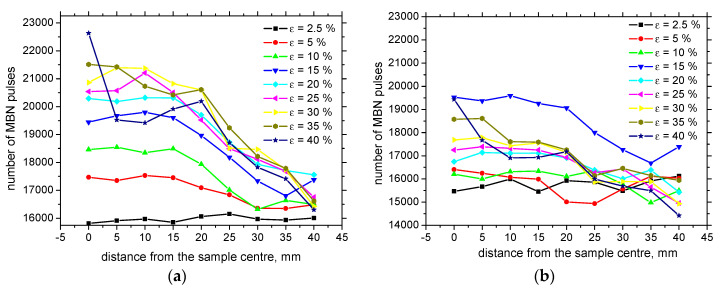
Number of MBN pulses versus *ε*. (**a**) RD; (**b**) TD.

Figure 16 illustrates that the MBN pulse height distribution versus *ε* is strongly altered. Their magnitude progressively decreases with *ε* at the expense of increasing number (see also Figure 17). However, the progressive increase in MBN pulse numbers is more apparent for the RD as compared to the TD. Figure 16 also shows that the stronger pulses can be found for the RD and the low *ε* (as compared with the TD, see Figure 16a). However, this relationship is inverse for the higher *ε* (see Figure 16b) as a result of the aforementioned shift of the easy axis of magnetization from the RD to TD. The number of MBN pulses decreases along with the sample length, and this decrease is less manifest for the TD.

The effective value (*rms*) of MBN is driven by the synergistic effect of MBN pulses number *n* and their magnitude *X_i_*, as follows:

(1) $$rms=\sqrt{\frac{1}{n}\overset{n}{\underset{i=1}{\sum }}X_{i}^{2}}$$

Decreasing MBN versus *ε* indicates that the increasing number of MBN pulses takes only a minor role and their reduced magnitude predominates. It is considered that the increasing number of MBN pulses at the expense of their reduced magnitude is driven by dislocation cells. These cells produce sub-grain boundaries in the grain interior and tend to refine the DWs alignment. DWs at 180° tend to be denser at the expense of their reduced length, and the increasing density of 90° oriented DWs are pinned by grain and sub-grain boundaries.

## 4. Discussion

**Figure 18:**
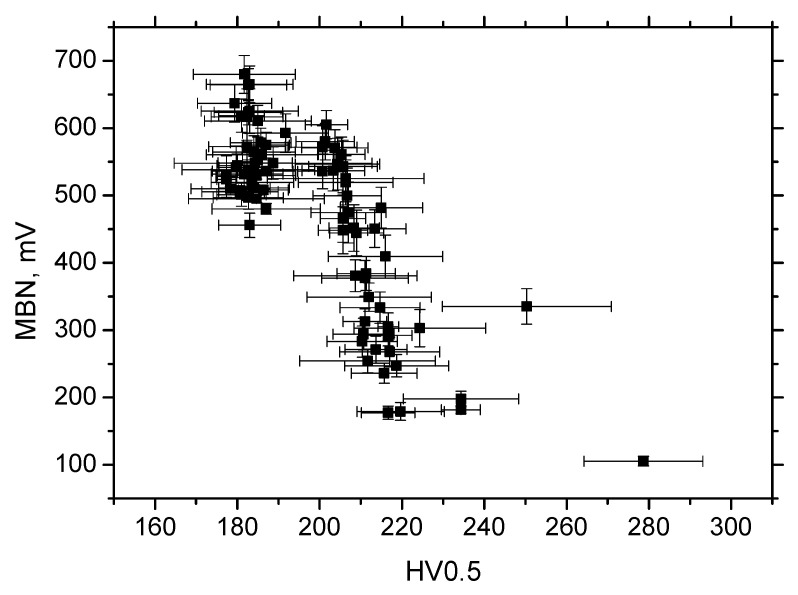
MBN versus HV0.5, RD.

**Figure 19:**
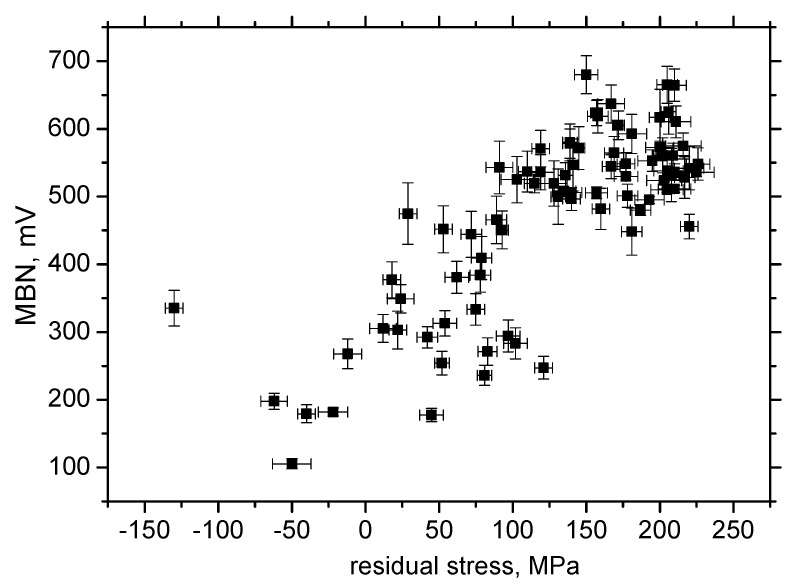
MBN versus residual stresses, RD.

It is clear that MBN is a function of residual stress as well as the superimposing microstructure (altered dislocation density). Figure 18 demonstrates that MBN shows a steep decrease versus HV0.5 due to interference of the DWs with dislocation cells, which pin DWs in their position, as well as reducing their free path of motion. On the other hand, this evolution tends to become moderate for the *ε* = 40% (in the center of the sample). Furthermore, the aforementioned DWs refinement also contributes to MBN. Figure 19 depicts the contribution of residual stresses to MBN, which proves the well-known relationship when compressive stresses tend to decrease the magnitude of MBN pulses and vice versa. Comparing Figure 7, Figure 9 and Figure 12, it can be seen that the correlation of MBN versus microhardness is stronger as compared with the correlation of MBN versus residual stress. The MBN and HV0.5 remain nearly constant versus sample length in the homogenous phase of plastic straining, whereas the residual stress profiles exhibit a progressive shift towards the bulk tensile stresses. Furthermore, the stress profiles for the RD exhibit the maximum outside the sample center, whereas MBN and microhardness do not.

The results of residual stress measurements show that these stresses in the near-surface region are shifted towards compressive stresses along with increasing plastic straining in RD (see Figure 9). It is considered that the compressive tensile stresses (for example for *ε* = 40%) produced by plastic straining are balanced (rearranged) by tensile stresses in the deeper layers (and vice versa). However, these stresses in the deeper regions are not affecting MBN due to limited skin depth of MBN (about 200 ÷ 300 µm). Despite the remarkable difference between XRD and MBN sensing depths, these techniques still can be considered as the near-surface techniques especially with respect of the sample thickness, which varies from 7.84 to 5.66 mm (see Table 2). Finally, it should be noticed that the contribution of residual stresses into evolution of MBN is limited (minor) and evolution of microstructure usually predominates in the case of Fe alloys [16,18,24]. Bozoroth [26] also found that the influence of lattice defects in iron-alloys usually predominates over the stress state. The Néel [27] equation can be applied for the assessment of the stress state and microstructure contribution to the coercive force as follows:

(2) $$H_{c}=K_{1}\times \alpha +K_{2}\times v$$

where *α* is the volume fraction of inclusions and *v* is the fraction of the material that is subjected to a large disturbing stress. Néel [27] calculated that K_1_ for iron is 360 and K_2_ is only 2.1, which indicates that the pre-magnetization process is more microstructurally sensitive, and the contribution of the stress state is limited.

**Figure 20:**
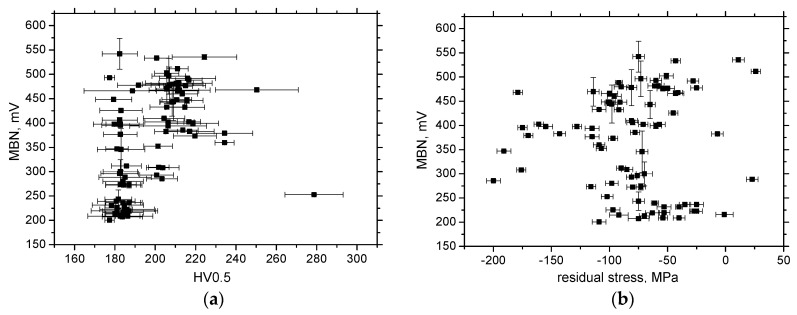
Correlations for MBN, TD. (**a**) MBN versus HV0.5; (**b**) MBN versus residual stresses.

Figure 20 illustrates that the correlation of MBN in the TD versus HV0.5, as well as residual stress, is missing, and this direction is not suitable for the assessment of the sample state (expressed in terms of microhardness and stress state) exposed to the developed *ε*.

**Figure 21:**
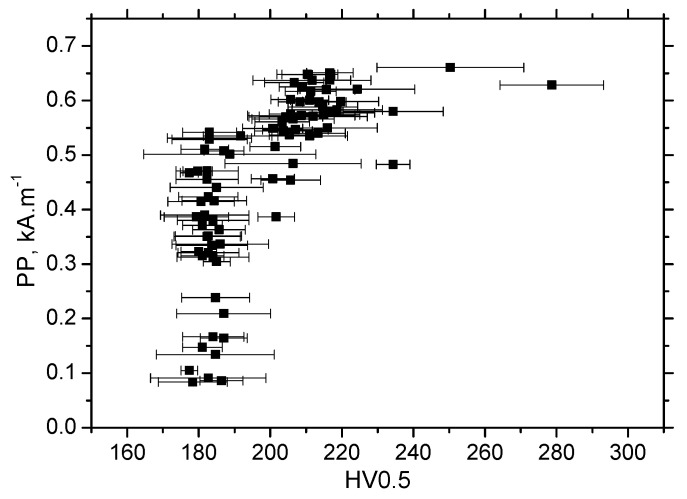
*PP* versus HV0.5, RD.

**Figure 22:**
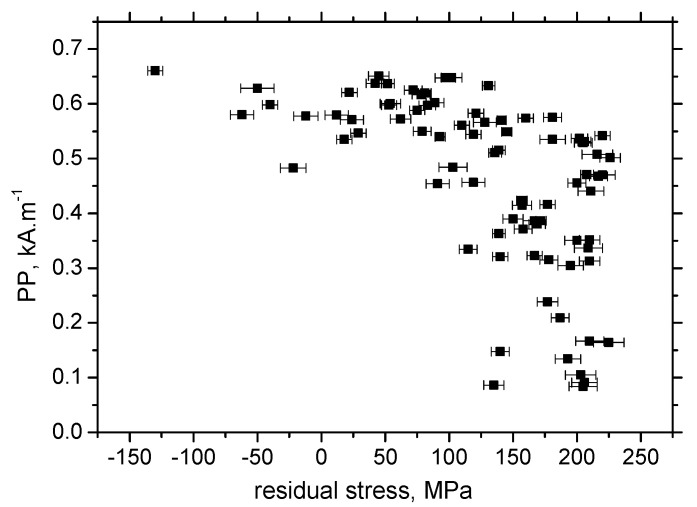
*PP* versus residual stresses, RD.

**Figure 23:**
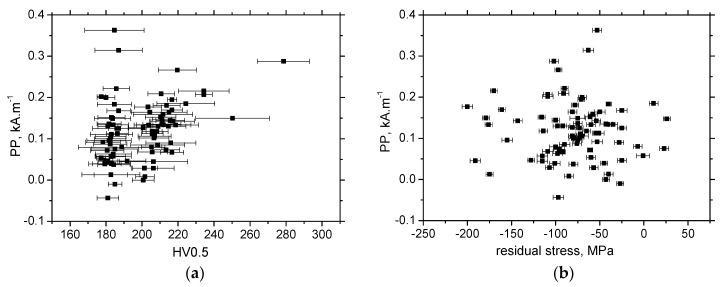
Correlations for *PP*, TD. (**a**) *PP* versus HV0.5; (**b**) *PP* versus residual stresses.

The evolution of *PP* versus HV0.5 exhibits quite a wide range of *PP* corresponding to the nearly untouched microhardness (as compared with the bulk, Figure 21). As soon as the plastic straining takes place and the dislocation density is altered, *PP* increases versus HV0.5 (due to the higher pinning strength of dislocation cells against DWs [14,23,24]), but this relationship saturates for the highest HV0.5. In addition, the correlation between *PP* and residual stresses (Figure 22) demonstrates quite poor sensitivity, since the relationship between residual stresses and *PP* in the region of compressive stresses up to approx. tensile stress of 75 MP, is too flat. The neighboring region of tensile stresses exhibits strongly varying *PP* values. Furthermore, Figure 23 depicts the missing correlation of *PP* versus HV0.5 (Figure 23a) as well as *PP* versus residual stresses (Figure 23b) in the TD. For these reasons, *PP* as a parameter extracted from the MBN signal cannot be employed for the assessment of the degree of the sample’s over-stressing on the unloaded bodies made of S235 steel.

## 5. Conclusions

The results of this study can be summarized as follows:

1. Low MBN alerts incoming rupture of S235 steel;
2. Distribution of MBN along the sample length is non-homogenous in the region of localized plastic strain, and the lowest MBN values can be found at the position of necking;
3. A more or less homogenous distribution of MBN is typical for homogenous plastic strains;
4. Due to the preferential elongation of the matrix during loading, the RD direction exhibits higher values as comparted with the TD; however, the easy axis of magnetization tends to turn into the TD for the higher *ε*;
5. MBN is a function of residual stresses as well as microhardness in the RD;
6. Sensitivity of *PP* against developed *ε* is lower as compared with MBN, and the correlation of *PP* versus HV0.5, as well as residual stress, is weak.

Finally, MBN could be considered as a promising method for non-destructive monitoring of ship and other components made of S235 steel with respect of their over-stressing and/or incoming rupture.

## Figures and Tables

**Table 1:** Chemical composition of S235 steel in wt.%.

Fe	C	Mn	Si	P	S
balance	0.22	1.6	0.05	0.05	0.05

**Table 2:** Engineering stress *σ*, true stress *σ_true_*, and *T*, *W*, *L* values for the different elastic and plastic strains.

Parameter	Elastic Deformation	Plastic Deformations							
		Homogenous Plastic Straining					Non-Homogenous (Localized) Plastic Straining—Necking		
*ε*, %	2.5	5	10	15	20	25	30	35	40
*T*, mm	7.84	7.78	7.65	7.48	7.30	7.13	6.84	6.45	5.66
*W*, mm	12.41	12.30	12.06	11.84	11.44	11.31	10.81	10.27	8.96
*L*, mm	49.80	50.40	52.00	53.80	56.03	57.93	59.67	61.38	63.61
*σ*, MPa	156	284	349	376	388	392	391	381	350
*σ_true_*, MPa	156	289	368	413	452	475	515	560	672

Notes: Strain *ε* represents the ratio between the sample elongation Δ*L* = *L* − *L*_0_ and initial length *L*_0_ (*ε* = Δ*L*/*L*_0_). Engineering stress *σ* is calculated by the use of the nominal cross sectional area of the samples as that measured before samples loading. True stress *σ_true_* is calculated by the use of the true cross sectional area of the samples (*T* × *W*) measured after samples loading.
